# Platelet volume indices have low diagnostic efficiency for predicting bone marrow failure in thrombocytopenic patients

**DOI:** 10.3892/etm.2012.799

**Published:** 2012-11-05

**Authors:** RUI-LONG XU, ZHAO-JING ZHENG, YONG-JUN MA, YING-PING HU, SHUN-HONG ZHUANG

**Affiliations:** 1 Department of Laboratory Medicine, Jinhua Municipal Central Hospital, Jinhua, Zhejiang 321000;; 2 School of Laboratory Medicine, Wenzhou Medical College, Wenzhou, Zhejiang 325027, P.R. China

**Keywords:** thrombocytopenia, platelet volume indices, mean platelet volume, platelet distribution width, idiopathic thrombocytopenia purpura, bone marrow failure

## Abstract

The aim of the present study was to evaluate the predictive efficiency of mean platelet volume (MPV) and platelet size deviation width (PDW) for bone marrow failure (BMF) in thrombocytopenic patients. Platelet count, MPV and PDW data were retrieved from the records of 574 unselected thrombocytopenic patients from between March 2010 and March 2011, of which 182 patients with a platelet count <20×10^9^/l were excluded from further study. A total of 392 valid thrombocytopenic patients were included in the present study and divided into two groups: 124 patients with idiopathic thrombocytopenia purpura (ITP) and 268 with BMF. The predictive efficiency of MPV and PDW were tested for the diagnosis of BMF. Significant differences were observed in the age distribution, platelet count, MPV and PDW between the ITP and BMF groups. The platelet count was positively correlated with MPV and PDW in the patients with ITP but not BMF. The negative-predictive values of MPV and PDW for BMF were 59.3 and 58.9%, respectively, with an MPV threshold of ≥11.0 fl and a PDW threshold of <16.0%. The positive-predictive values of MPV and PDW for BMF were 88.4 and 83.9%, respectively, with an MPV threshold <8.0 fl and a PDW threshold ≥17.5%. The areas under the curves (AUCs) of MPV and PDW were 0.281 and 0.700, respectively, for the diagnosis of BMF. The negative and positive-predictive values of MPV for BMF at different thresholds were not as conclusive as described in previous studies. MPV and PDW do not have sufficient specificity and sensitivity for the diagnosis of BMF in thrombocytopenic patients.

## Introduction

Platelet volume indices (PVIs) are a group of parameters which are inexpensive to measure and are derived from routine blood counts. The mean platelet volume (MPV) and platelet deviation width (PDW) are the best validated and prominent of these and are attractive indices for research in clinical settings due to their widespread availability to clinicians (1–3). While data on the size (mean cell volume, MCV) and deviation width (RDW) of red blood cells are universally used for the investigation of anemia, the diagnostic value of PVIs in thrombocytopenia has not been fully established. The MPV has been demonstrated to be sensitive and specific in discriminating between thrombocytopenia caused by idiopathic thrombocytopenia purpura (ITP) and by aplastic anemia (4). The MPV may markedly correlate with the presence or absence of bone marrow diseases in thrombocytopenic patients (5). A prospective evaluation has revealed that the mean MPV of normal Thai individuals may be used as a cut-off value (7.9 fl) in distinguishing hyperdestructive from hypoproductive thrombocytopenia (6). However, it has been reported that although MPV may be used as an initial suggestion of bone marrow disease in thrombocytopenic patients, it has limited sensitivity and specificity (7). It has also been suggested that MPV and PDW are not useful for identifying the mechanism of chemotherapy-induced thrombocytopenia in a population of adult oncology patients (8). Furthermore, there are few studies evaluating the diagnostic power of PVI for discriminating thrombocytopenia caused by bone marrow failure (BMF) from that caused by ITP in Chinese patients at present. To explore the clinical usefulness of MPV and PDW in predicting the presence of bone marrow disease in thrombocytopenic patients, a retrospective evaluation of MPV and PDW in a standard clinical setting of a tertiary teaching hospital was conducted.

## Materials and methods

### Subjects

Based on the clinical and laboratory information, the thrombocytopenic patients were divided into two groups: those with BMF (361 patients) and those with no evidence of BMF (213 patients). When platelet counts were <20×10^9^/l, the resultant PVI, in particular MPV, may exhibit significant discrepancies (9). Considering this fact, those patients with a platelet count <20.0×10^9^/l were excluded from further study. The presence of bone marrow disease was determined with the aid of a bone marrow examination in the 268 valid patients with BMF and the 124 with ITP. Two morphologists (Y.P. Hu and S.H. Zhuang) reported on the bone marrow samples independently.

### Instruments and quality control

EDTA anti-coagulated venous blood samples were collected from patients attending the Department of Hematology at the Jinhua Municipal Central Hospital (Zhejiang, China). The present study was conducted in accordance with the Declaration of Helsinki and with approval from the Ethics Committee of Jinhua Municipal Central Hospital. Written informed consent was obtained from all participants. Thrombocytopenia was defined as a platelet count of <100×10^9^/l. MPV was measured on a Beckman Coulter (Beckman-Coulter, Miami, FL, USA) Gen-S automated analyzer. Calibration was assessed daily with the commercial calibrant 5C (Beckman Coulter) and monitored twice daily.

### PVI measurement

Since no statistically significant changes have been demonstrated to occur in platelet and platelet indices when blood samples are stored at room temperature for 24 (5) and 48 h (10), all the whole blood counts were assayed within 4 h of the sample collection, although not at a fixed time-point. In addition, the pre-analytical variances, such as the storage time after sample collection, were not considered further in the present study.

### Statistical analysis

All statistical evaluations were performed with the SPSS 13.0 software package. P<0.05 was considered to indicate statistically significant differences.

## Results

### Clinical characteristics and platelet count

In total, 574 cases of thrombocytopenic patients with platelet counts <100×10^9^/l were retrieved from the records dating from between March 2010 and March 2011. According to the diagnoses, the 574 cases were divided into two groups: ITP- (n=213) and BMF-associated thrombocytopenia (n=361). A total of 89 of the 213 patients assigned to the ITP group and 93 of the 361 patients assigned to the BMF group had platelet counts <20×10^9^/l. Since the resultant platelet indices, in particular the MPV, are less reliable when the platelet count is <20×10^9^/l, these patients were excluded. However, it was observed that the percentage of platelet counts <20×10^9^/l differed significantly (P<0.01) between ITP (41.8%) and BMF (25.8%).

Table I shows the diagnoses for thrombocytopenic patients who had platelet counts ≥20.0×10^9^/l. The clinical and laboratory characteristics of the patients in the ITP and BMF groups are presented in Table II. Statistically significant differences were observed in the MPV, PDW, platelet count and age between the two groups of patients. Similar to the results of previous studies, no significant differences were observed in the gender distribution between the ITP and BMF groups.

### Correlation between PVI and platelet count

**Figure 1:**
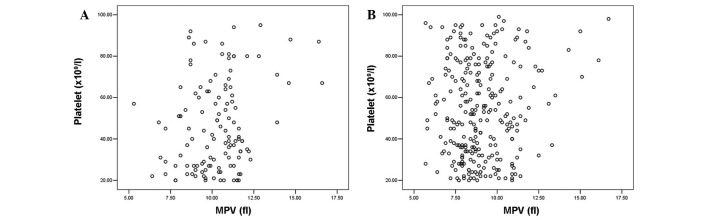
Scatter plots for (A) patients with ITP (n=124) and (B) patients with BMF (n=268) showing platelet count vs. MPV. A positive correlation between platelet count and MPV was observed in patients with ITP (r=0.248, P=0.005) but not in patients with BMF (r=0.097, P=0.112). ITP, idiopathic thrombocytopenia purpura; BMF, bone marrow failure; MPV, mean platelet volume.

**Figure 2:**
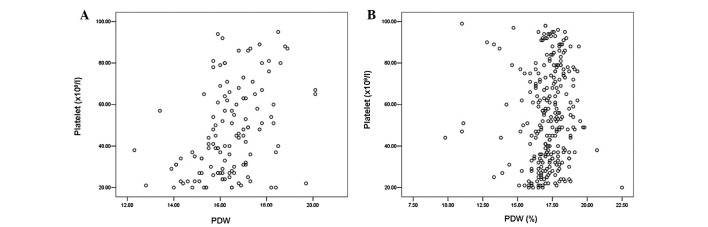
Scatter plots for (A) patients with ITP (n=124) and (B) patients with BMF (n=268) showing platelet count vs. PDW. A positive correlation between platelet count and PDW was observed in patients with ITP (r=0.389, P=0.000) but not in patients with BMF (r=−0.017, P=0.778). ITP, idiopathic thrombocytopenia purpura; BMF, bone marrow failure; PDW, platelet deviation width.

Fig. 1 shows the scatter plots of platelet count against MPV for (A) patients with ITP and (B) patients with BMF. Unlike the patients with BMF, a positive correlation between the platelet count and MPV was revealed in patients with ITP (r=0.248, P=0.005). Notably, a positive correlation between the platelet count and PDW was also demonstrated (Fig. 2) in the ITP group (r=0.389, P=0.000) but not in the BMF group (r=−0.017, P=0.778).

### Predictive efficiency of MPV and PDW

The functions of MPV and PDW for the diagnosis of BMF were evaluated in thrombocytopenic patients in the present study. It was revealed that the level of MPV did not predict the risk of BMF for the thrombocytopenic patients. Table III shows the correlation between MPV and its negative-predictive value for BMF. An MPV of ≥11.0 fl has a negative-predictive value of 59.0% and may be applied in 20.7% of patients.

A low MPV is associated with a higher risk of BMF in thrombocytopenic patients. Table IV shows the correlation between the MPV and the positive-predictive value for BMF at various cut-off values of MPV. An MPV of <8.0 fl has the highest positive-predictive value for BMF (88.4%) and may be applied in 21.9% of patients with low platelet count.

The correlation between PDW and its predictive efficiency for BMF in thrombocytopenic patients in the present study is summarized in Tables V and VI. A PDW of ≥19.0% has the highest positive-predictive value for BMF in thrombocytopenic patients in the present study but may only be applied in 5.4% of the study patients. A PDW of ≥17.5% has a positive-predictive value of 83.9% and may be applied to 36.5% of these patients. By contrast, a PDW of <16.0% has the highest negative-predictive value (58.9%) for BMF and may be applied to 18.6% of thrombocytopenic patients.

**Figure 3:**
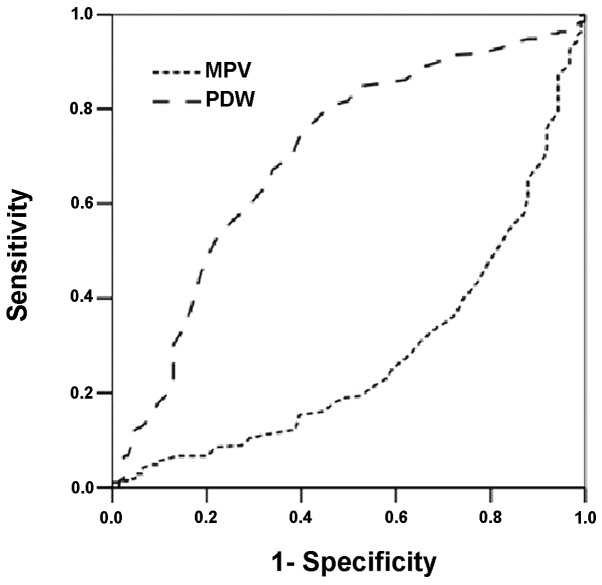
ROC curves of MPV and PDW for diagnosis of BMF in thrombocytopenic patients. ROC, receiver operating characteristic; MPV, mean platelet volume; PDW, platelet deviation width; BMF, bone marrow failure.

The areas under the curve (AUCs) of MPV and PDW in receiver operating characteristic (ROC) analysis were 0.281 and 0.700, respectively, for the diagnosis of BMF in thrombocytopenic patients in the present cohort (Fig. 3).

## Discussion

Thrombocytopenia is a common clinical manifestation of many diseases and has numerous causes, including decreased bone marrow production, increased spleen sequestration and accelerated destruction of platelets (11). One of the major causes of increased platelet destruction is immune thrombocytopenia, in which autoantibodies bind to platelet antigens, causing their premature destruction by the reticular-endothelial system, particularly the spleen (12). BMF, including hematological malignancy, tumor infiltration and aplastic anemia, is another common cause of thrombocytopenia and its diagnosis requires confirmation by hematological morphology, bone marrow examination, immunophenotyping and karyotyping which are familiar only to hematologists.

Rapid diagnostic blood tests are used routinely in clinical practice to assess the risk of specific underlying diseases in groups of patients presenting a defined set of symptoms or test results.

Although platelet parameters, such as MPV and PDW, have been available for some time, their clinical usefulness has been elusive (13), in particular due to the delay between blood collection and analysis.

The results of the present study revealed that patients with BMF have higher platelet counts, lower MPV and higher PDW than patients with ITP. The reduced production of platelets in patients with ITP involves more platelets being consumed by the reticular-endothelial system and this higher turnover is in turn reflected by the lower platelet counts and higher MPV (14,15). PDW is measured using an automated blood analyzer based on a logarithmic transformation of the platelet count. A higher PDW in patients with BMF is consistent with a significant dysplasia of hematopoiesis in the bone marrow.

MPV has been evaluated as a diagnostic tool in different conditions with thrombocytopenia with contradictory results. It has been demonstrated that MPV has sufficient sensitivity and specificity to discriminate aplastic anemia (4), bone marrow disease (5), hypoproductive thrombocytopenia (6) and bone marrow metastasis of solid tumors (16) from immune-associated thrombocytopenia. Furthermore, it has been reported that MPV and PDW may be safely relied on for a positive diagnosis of ITP. MPV and PDW were superior to the platelet large cell ratio (17). MPV also has clinical benefits for the diagnosis and/or prognosis of Crimean-Congo hemorrhagic fever (18), inherited macrothrombocytopenias (19) and cardiovascular and pulmonary embolism (20).

However, it has been reported that although MPV may be used as an initial suggestion of bone marrow disease in thrombocytopenic patients, it has limited sensitivity and specificity (7). It has also been suggested that MPV and PDW are not useful for identifying the mechanism of chemotherapy-induced thrombocytopenia in a population of adult oncology patients (8). Similarly, MPV cannot be used to identify the etiology of thrombocytopenia in the babies of preeclamptic mothers (21). The results of the present study revealed that, although there are significant differences in the MPV and PDW between BMF and ITP, the two parameters do not have enough sensitivity and specificity to predict the presence of bone marrow disease in thrombocytopenic patients.

In the preliminary phase of the present study, the incidence of platelet counts <20×10^9^/l was observed to be higher in the present series of thrombocytopenic patients (182/574, 31.7%) than that reported by Bowles *et al* (58/473, 12.3%) (5). The percentage of thrombocytopenic patients with platelet counts <20×10^9^/l was higher in patients with ITP than in the patients with BMF (89/213, 41.8% vs. 93/361, 25.8%; P<0.01). Methodologically speaking, the validity of MPV and PDW is negatively affected by a platelet count <20×10^9^/l. Although Bowles *et al* reported that the MPV retained a diagnostic predictive value at platelet counts ≤20×10^9^/l (5), the thrombocytopenic patients with platelet counts <20×10^9^/l were not included in the present study for further analysis. It is unclear whether patients with platelet counts <20×10^9^/l were included in calculating the diagnostic predictive efficiency of MPV and PDW in other reports and this may be one of the reasons for the discrepancies between various authors. Considering the relatively high prevalence of thrombocytopenia with platelet counts <20×10^9^/l, further studies should be carried out which include patients with platelet counts <20×10^9^/l to elucidate the possible impact on the overall predictive efficiency of MPV and PDW for the diagnosis of BMF in thrombocytopenic patients.

As noted by Leader *et al*, the majority of these data on the diagnostic predictive efficiency of MPV and PDW in thrombocytopenic patients are from retrospective studies, some of which had small study populations and confounding factors influencing platelet volume. Additionally, the cut-off values derived from these retrospective studies have not been validated prospectively (20). In future, improved research designs and standardized PVI measurements may significantly increase the diagnostic predictive power of MPV and PDW in the differential diagnosis of thrombocytopenia.

In conclusion, significant differences were observed in platelet count, MPV, PDW and patient age between patients with ITP and patients with BMF. However, MPV and PDW do not have enough predictive efficiency for the diagnosis of BMF in thrombocytopenic patients.

## Figures and Tables

**Table I:** Underlying diseases of the thrombocytopenic patients with platelet count ≥20.0×10^9^/l but <100×10^9^/l.

Diagnosed cause of thrombocytopenia	Number of patients (%)
Bone marrow failure	268
Acute lymphocytic leukemia	28 (10.4)
Acute myelogenous leukemia	118 (44.1)
Myelodysplasia syndrome	30 (11.2)
Aplastic anemia	24 (9.0)
Metastatic malignancy	11 (4.1)
CML blast crisis	17 (6.3)
Plasmocytic dyscrasia	34 (12.7)
Lymphoma	6 (2.2)
Idiopathic purpura thrombocytopenia	124
Total	392

CML, chronic myelogenous leukemia.

**Table II:** Clinical and laboratory characteristics in the groups of patients with thrombocytopenia.

Characteristic	ITP (n=124), mean ± SD	BMF (n=268),mean ± SD	P-value
Age (years)	39.6±22.9	49.4±20.0	0.000
Gender (M/F)	65/59	135/133	0.706
Platelets (×10^9^/l)	46.5±21.4	53.7±22.9	0.003
MPV (fl)	10.3±1.8	9.0±1.8	0.000
PDW (%)	16.4±1.4	17.3±2.3	0.001

ITP, idiopathic thrombocytopenia purpura; BMF, bone marrow failure; MPV, mean platelet volume; M, male; F, female; PDW, platelet deviation width.

**Table III:** Comparison of the negative-predictive value of the MPV for bone marrow diseases at various MPV values.

MPV threshold (fl)	Number of patients	Number of patients without bone marrow disease	% of all patients within this group	Negative-predictive value given MPV (%)	Odds ratio	95% CI
≥11.0	81	48	20.7	59.3	0.222	0.133–0.371
≥10.5	112	61	28.6	54.5	0.243	0.152–0.387
≥10.0	139	73	35.5	52.5	0.228	0.145–0.359
≥9.5	180	87	45.9	48.3	0.226	0.143–0.358
≥9.0	213	96	54.3	45.1	0.226	0.139–0.367
≥8.5	263	108	67.1	41.1	0.203	0.114–0.362
≥8.0	306	114	78.1	37.3	0.222	0.110–0.446
≥7.5	343	117	87.5	34.1	0.322	0.140–0.739

MPV, mean platelet volume; CI, confidence interval.

**Table IV:** Comparison of the positive-predictive value of the MPV for bone marrow diseases at various MPV values.

MPV threshold (fl)	Number of patients	Number of patients with bone marrow disease	% of all patients within this group	Positive-predictive value given MPV (%)	Odds ratio	95% CI
<11.0	311	235	79.3	75.6	4.498	2.692–7.514
<10.5	280	217	71.4	77.5	4.120	2.586–6.564
<10.0	253	202	64.5	79.8	4.381	2.785–6.891
<9.5	212	175	54.1	82.5	4.425	2.794–7.006
<9.0	179	151	45.7	84.4	4.425	2.724–7.189
<8.5	129	113	32.9	87.6	4.921	2.760–8.774
<8.0	86	76	21.9	88.4	4.513	2.243–9.077
<7.5	49	42	12.5	85.7	3.106	1.353–7.129

MPV, mean platelet volume; CI, confidence interval.

**Table V:** Comparison of the positive-predictive value of PDW for bone marrow disease at various PDW values.

PDW threshold (%)	Number of patients	Number of patients with bone marrow disease	% of all patients within this group	Positive-predictive value given PDW (%)	Odds ratio	95% CI
≥19.0	21	18	5.4	85.7	2.904	0.839–10.049
≥18.5	43	35	11.0	81.4	2.178	0.979–4.846
≥18.0	87	71	22.2	81.6	2.433	1.347–4.393
≥17.5	143	120	36.5	83.9	3.561	2.132–5.946
≥17.0	214	173	54.6	80.8	3.687	2.350–5.782
≥16.5	273	216	69.6	79.1	4.883	3.066–7.775
≥16.0	319	238	81.4	74.6	4.212	2.479–7.155

PDW, platelet distribution width; CI, confidence interval.

**Table VI:** Comparison of the negative-predictive values of PDW for bone marrow disease at various PDW values.

PDW threshold (%)	Number of patients	Number of patients without bone marrow disease	% of all patients within this group	Negative-predictive value given PDW (%)	Odds ratio	95% CI
<19.0	371	121	94.6	32.6	0.344	0.100–1.192
<18.5	349	116	89	33.2	0.459	0.206–1.021
<18.0	305	108	77.8	35.4	0.411	0.228–0.742
<17.5	249	101	63.5	40.6	0.281	0.168–0.469
<17.0	178	83	45.4	46.6	0.271	0.173–0.425
<16.5	119	67	30.4	56.3	0.205	0.129–0.326
<16.0	73	43	18.6	58.9	0.237	0.140–0.403

PDW, platelet distribution width; CI, confidence interval.
